# Evaluation of Automated Vasopressor Administration Algorithms Using Lower-Limit Control for Intraoperative Hypotension: A Simulation Study

**DOI:** 10.3390/jcm14186615

**Published:** 2025-09-19

**Authors:** Emi Morinushi, Osamu Nagata, Fumiyo Yasuma, Aya Kuroyanagi, Kanji Uchida

**Affiliations:** 1 Department of Anesthesiology and Pain Relief Center, The University of Tokyo Hospital, Tokyo 113-8655, Japan; uchidak-ane@h.u-tokyo.ac.jp; 2 Department of Anesthesiology and Reanimatology, University of Fukui Hospital, Fukui 910-1193, Japan; onagata@u-fukui.ac.jp (O.N.); fyasuma@u-fukui.ac.jp (F.Y.); 3 Department of Anesthesiology, Saitama Cooperative Hospital, Saitama 333-0831, Japan; ayatake@maple.ocn.ne.jp

**Keywords:** evaluation metric, lower-limit control, blood pressure, vasopressor, simulation

## Abstract

**Background/Objectives:** The aim of this study was to develop evaluation metrics for lower-limit vasopressor control, a strategy intended to prevent prolonged intraoperative hypotension under noninvasive blood pressure monitoring. **Methods:** Using general-purpose simulation software, we developed a blood pressure generation model with one-minute intervals and an automated vasopressor administration model with five-minute intervals. The latter delivered drugs according to predefined rules when systolic blood pressure (sBP) fell below a threshold. Four dosing strategies were constructed by combining bolus, repeated low-dose bolus, and continuous infusion approaches. Simulations were performed, and the following evaluation metrics were calculated: (1) proportion of time below threshold (PTBT), (2) mean value below threshold (MVBT), (3) average sBP, and (4) median performance error (MDPE) and median absolute performance error (MDAPE). **Results**: PTBT and MVBT analyses showed that incorporating continuous infusion reduced both the duration and severity of hypotension. Moreover, adding MVBT to the average sBP after subtracting the threshold quantified the extent to which sBP exceeded the threshold on average. In contrast, MDPE and MDAPE varied substantially with the assumed target pressure, highlighting their limitations in evaluating lower-limit control without a fixed target. **Conclusions**: For lower-limit control, metrics such as PTBT, MVBT, and average sBP offer useful insights into control stability and hypotension avoidance, whereas MDPE and MDAPE may be unsuitable for quantitative assessment when the primary goal is to exceed a threshold rather than achieve a fixed target pressure.

## 1. Introduction

Intraoperative hypotension is a well-recognized risk factor for postoperative cardiac and cerebrovascular events, acute kidney injury [1,2,3], and increased mortality [4]. Prompt and appropriate management can mitigate postoperative organ dysfunction [5] and reduce medical expenses [6].

Conventional management strategies include the administration of vasopressors and fluid therapy. However, clinical decision-making during hypotensive episodes often relies heavily on empirical judgment, which may cause delays and increase anesthesiologists’ workloads across diverse clinical settings. Automated vasopressor delivery systems have been developed to enable faster and more precise responses. Most proposed control strategies for automated vasopressor administration [7,8,9,10,11,12,13,14] use proportional–integral–derivative (PID) control to maintain blood pressure near a target value. In contrast, anesthesiologists’ manual practices often follow a “lower-limit control” approach—administering vasopressors only when blood pressure falls below a predefined threshold. This simple, intuitive method has demonstrated clinical feasibility, particularly with phenylephrine-based interventions [15].

Automation is not intended to replace anesthesiologists but rather support them. Algorithm-based systems can reduce repetitive tasks and improve responsiveness to hypotensive episodes. Despite its simplicity, lower-limit control is most often used alongside intermittent noninvasive blood pressure (NIBP) monitoring. Unlike PID-based systems, however, no standardized framework exists for reproducibly evaluating blood pressure control performance under lower-limit control.

This study aimed to evaluate the performance of vasopressor administration algorithms based on lower-limit control by (1) modeling blood pressure response to vasopressor administration; (2) simulating algorithm behavior in response to intermittent blood pressure measurements under predefined rules; and (3) identifying evaluation indices that effectively characterize control accuracy. We propose a methodology for assessing vasopressor administration algorithms for intraoperative hypotension under noninvasive blood pressure monitoring that provides a general framework independent of specific blood pressure thresholds or particular vasopressors.

## 2. Materials and Methods

This study was conducted entirely in silico; therefore, ethical approval was not required.

### 2.1. Simulation Software

We used the commercially available simulation software ExtendSim^®^ CP v10.1.0 (ANDRITZ Inc., Decatur, GA, USA), a modeling platform that allows users to build, run, and analyze simulations in a visual environment. Models are created using a drag-and-drop interface, and parameters are set for each block to execute simulations. Programming can also be incorporated to create components with custom functions.

### 2.2. Simulation Framework

This simulation framework consisted of two components: a blood pressure (BP) generation module and a vasopressor dosing control module.

In the BP generation module, the vasopressor dose served as the input, and the output was the corresponding change in blood pressure (ΔBP). Adding ΔBP to the baseline systolic blood pressure (baseline sBP) yielded the generated systolic blood pressure (generated sBP).

In the vasopressor dosing control module, the vasopressor dose was calculated according to predefined control algorithms. Both the repeated bolus dose and the continuous infusion rate were provided as inputs to the BP generation model.

**Figure 1:**
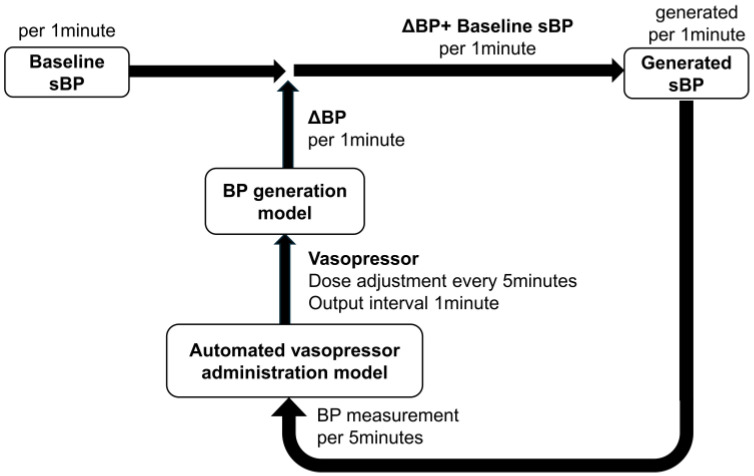
Simulation configuration.

An overview of the simulation configuration is shown in Figure 1.

### 2.3. Baseline Systolic Blood Pressure (sBP)

**Figure 2:**
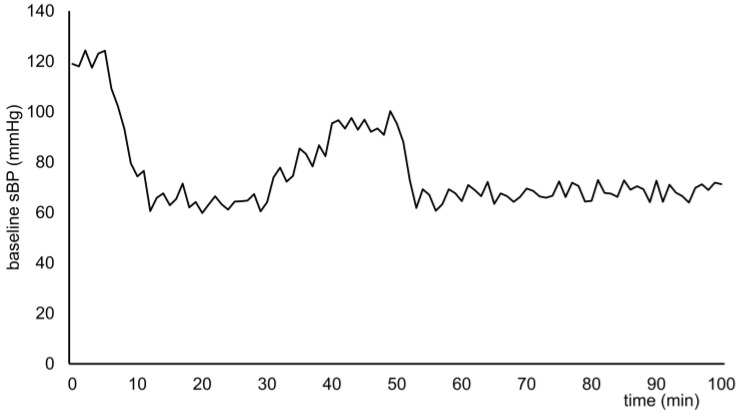
Baseline systolic blood pressure (baseline sBP). This baseline sBP represents one example used in the simulation. For each run, a uniform random error ranging from –5 to +5 mmHg was added to the baseline sBP value to introduce variability.

Any blood pressure parameter—such as systolic blood pressure (sBP) or mean blood pressure—can be selected as the control target. However, due to its clinical relevance and frequent use in intraoperative hemodynamic monitoring, sBP was chosen as the reference parameter. Virtual baseline sBP data were generated at one-minute intervals over 100 min, assuming relatively stable conditions with a need for vasopressor support. These data included clinically typical hypotensive phases: initial hypotension due to anesthetic-induced vasodilation, transient hypertensive responses to surgical stimulation, and sustained hypotension. The baseline sBP variation is illustrated in Figure 2. This hemodynamic profile was designed to simulate a typical clinical scenario in which vasopressor administration is frequently required from anesthetic induction through the early surgical phase. For each simulation, random error fluctuations ranging from –5 to +5 mmHg were added to the baseline blood pressure.

### 2.4. Model Building

We developed two models: a blood pressure (BP) generation model to estimate changes in BP following vasopressor administration and a vasopressor administration model that outputs vasopressor dosage based on predefined algorithms.

**BP generation model**

**Figure 3:**
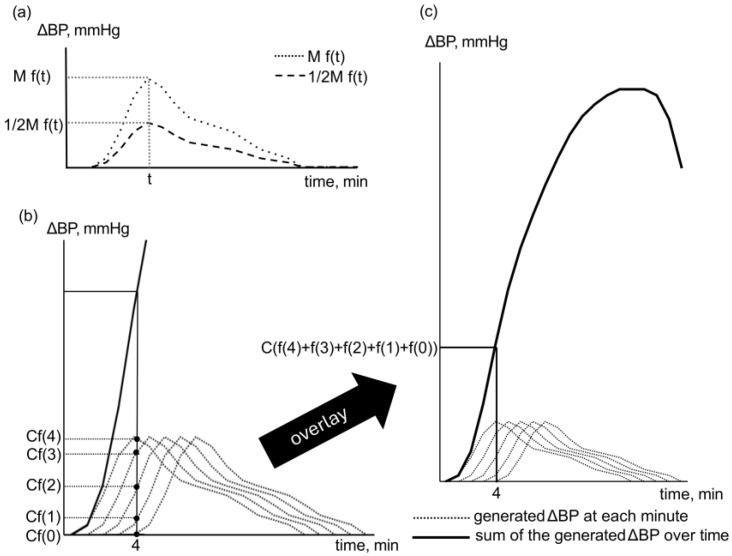
(**a**) BP elevation over time following bolus administration of a specific vasopressor at doses M (mg) and ½M (mg). (**b**) BP elevation over the first four minutes of continuous administration at rate C [mg/min], showing the five ΔBP waveforms generated from minute 0 to minute 4. (**c**) Cumulative BP elevation derived from the ΔBP waveforms in (**b**), represented as a thick black line.

The BP generation model receives vasopressor doses at one-minute intervals and outputs the corresponding change in blood pressure (ΔBP) each minute. This ΔBP is added to the baseline sBP to yield the generated sBP, which is used as an evaluation metric. The model calculates ΔBP under different dosing conditions as follows:

- Blood pressure elevation data from small doses of a specific vasopressor are used to construct the function of blood pressure increase over time (ΔBP = f(t)), where t is the elapsed time in minutes after administration.
- The elevation in BP over time following a bolus dose M [mg] is expressed as the waveform ΔBP = Mf(t) (Figure 3a).
- Assuming a linear dose–response relationship, administering half the dose (½ M [mg]) produces a waveform with half the effect, represented as ½ $Mf\left(t\right)\;$ (Figure 3a).
- For continuous administration at a constant rate C [mg/min], the waveform ΔBP = Cf(t) is generated every minute. Cumulative BP elevation at t minutes after starting continuous administration is expressed as the sum of t + 1 waveforms.
- For constant continuous administration at rate C [mg/min], BP elevation at t min after starting continuous administration is expressed as $\sum_{0}^{t}Cf(t)$. For example, BP elevation at 4 min after initiating continuous administration at rate C [mg/min] is expressed as ΔBP = $Cf\left(4\right)+Cf\left(3\right)+Cf\left(2\right)+Cf\left(1\right)+Cf(0)$, corresponding to the sum of five waveforms (Figure 3b). The transition of BP elevation during continuous administration is shown in Figure 3c.
- At time t [min], ΔBP is calculated by cumulatively summing the contributions from each waveform generated at one-minute intervals up to t minutes. This ΔBP is then added to the baseline sBP to obtain the generated sBP. In each simulation run, a random error variation of ±5% is applied to ΔBP.

**The vasopressor administration model**

The vasopressor administration model receives the generated sBP from the BP generation model at 5 min intervals. When sBP falls below 85 mmHg, the model calculates the vasopressor dose to be administered every minute over the next five minutes according to a predefined control algorithm. In this study, four dosing strategies—(A), (B), (C), and (D)—were implemented to reflect common clinical practices. All four algorithms are triggered when sBP drops below 85 mmHg. The lower limit of sBP 85 mmHg used in this study is not a universally validated optimal target but was selected as an illustrative example. The four dosing strategies are described as follows:

- Algorithm (A) administers a fixed dose M (mg) repeatedly whenever sBP < 85 mmHg.
- Algorithm (B) administers a fixed dose M (mg) upon the first occurrence of sBP < 85 mmHg, followed by a fixed half-dose of M/2 (mg) for subsequent occurrences.
- Algorithm (C) follows the same dosing protocol as (B) but combines it with continuous infusion starting at the time of the second administration.
- Algorithm (D) is similar to (C), but continuous infusion is suspended when the generated sBP exceeds 105 mmHg.

For algorithms (C) and (D), the initial continuous infusion rate is calculated based on the interval between preceding bolus administrations. Thereafter, the infusion rate is adjusted at each evaluation point according to a predefined rule based on the generated sBP.

### 2.5. Simulation Setup

Simulations were conducted over 100 min using discrete one-minute time steps (i.e., one step = one minute). A single baseline sBP profile was used for all runs, with random variation added independently in each run: –5 to +5 mmHg to the baseline sBP and –5% to +5% to ΔBP. For each algorithm ((A), (B), (C), and (D)), 10 simulation runs were performed. In each run, the generated sBP was recorded at 1 min intervals, and evaluation metrics were calculated accordingly.

### 2.6. Evaluation Metrics and Analysis Method

**Figure 4:**
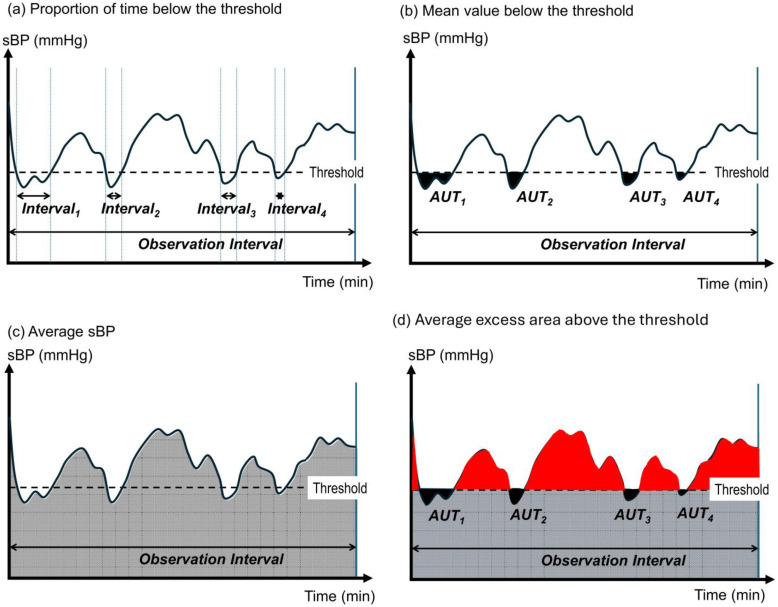
Composite visualization of metrics used to evaluate sBP control performance. Time (minutes) is plotted on the *x*-axis and sBP (mmHg) on the *y*-axis. (**a**) Proportion of time below the threshold (PTBT), defined as the cumulative duration during which sBP falls below the threshold (Intervals 1–4) divided by the total observation period, expressed as a percentage. (**b**) Mean value below threshold (MVBT). During periods when sBP is below the threshold, the cumulative difference between sBP and the threshold is illustrated as the black-shaded area (area under the threshold, AUT). MVBT is calculated as AUT divided by the observation period and reflects the average magnitude of deviation below the threshold. (**c**) Average sBP, represented as the gray-shaded area under the sBP curve divided by the observation period. (**d**) Average excess area above the threshold. The red-shaded area represents the cumulative extent to which sBP exceeds the threshold. Dividing this area by the observation period yields the average excess area above the threshold. This metric is derived as the sum of the gray-shaded area (average sBP) and the black-shaded area (MVBT), each normalized by the observation period and adjusted by subtracting the threshold level. It quantifies potential overtreatment due to excessive vasopressor administration.

To evaluate the control performance of algorithms (A) through (D), the following metrics were calculated based on sBP values recorded at one-minute intervals over the 100 min simulation. An overview of the metrics is illustrated in Figure 4.

- **Proportion of time below threshold (PTBT)**

PTBT represents the proportion of time during which sBP falls below the threshold of 85 mmHg (Figure 4a). In evaluating lower-limit control, it is important to determine whether this value is close to 0%.

$$PTBT\;(\%)=100\times \frac{\sum_{i=0}^{100}m_{i}}{observation\;period},m_{i}=\left\{\begin{array}{c} 1\;(sBPi\leq 85\;mmHg)\\ 0\;(sBPi>85\;mmHg) \end{array}\right.i\;=\;0,1,2\ldots ,100\;(\text{min})$$

- **Mean value below threshold (MVBT)**

MVBT represents the area under the threshold (AUT) during periods when sBP is below 85 mmHg, divided by the observation period. It reflects the average depth of sBP below the threshold (Figure 4b).

$$MVBT\left(mmHg\right)=\frac{the\;cumulative\;area\;of\;sBP\;values\;below\;the\;threshold\;of\;85\;mmHg}{observation\;period}$$

When PTBT is equivalent across groups, comparing MVBT values allows evaluation of the precision of vasopressor control algorithms.

- **Average sBP**

Average sBP represents the mean of the generated sBP over the observation period, capturing the central tendency of the distribution. When evaluated alongside MVBT, it quantifies the extent to which sBP exceeds the predefined threshold on average.

Average sBP = cumulative sBP ÷ observation period

The cumulative sBP is represented by the gray-shaded area in Figure 4c.

The average excess over the threshold can be computed as

(Average sBP + MVBT) − threshold value

that is,

(Average sBP + MVBT) − (85 mmHg × observation period ÷ observation period)

The cumulative area exceeding the threshold is represented by the red-shaded area in Figure 4d.

- **Median performance error (MDPE) and median absolute performance error (MDAPE)**

MDPE and MDAPE are non-parametric metrics characterizing deviations between the target blood pressure and the generated sBP during the observation period, expressed as percentages (%). MDPE indicates the median percentage deviation relative to the target, reflecting bias (whether generated values are generally above or below the target). However, it does not provide information about the distribution of larger deviations beyond the median.

MDAPE is defined as the median of the absolute percentage deviations from the target. It measures the magnitude of deviation regardless of direction and serves as an indicator of median error size. Since MDAPE corresponds to the 50th percentile of absolute deviations, approximately half of the deviations are smaller than this value. However, it provides no information about the remaining half, which may include extreme outliers, and this limitation should be noted.

In lower-limit control, the primary goal is to prevent excessively low blood pressure, making it difficult to define a specific target value. Therefore, in this study, MDPE and MDAPE were calculated using provisional target values of 85, 90, 95, 100, and 110 mmHg.

Percentage performance error (PE), MDPE, and MDAPE are calculated using the following formulas [16,17]:
PE*ij* denotes the *i*-minute data point in the *j*th simulation.PE*ij* (%) = (sBP*ij* − target sBP*ij*) × 100/target sBP*ij*
*i* = 0, 1, 2…, 100 (min); *j* = 1, 2, 3…, 10 (simulation number). 
MDPE*j* and MDAPE*j* are the values for the *j*th simulation, based on the median of PE*ij*.MDPE*j* and the MDAPE*j* are the data for the *j*th simulation.MDPE*j* (%) = median {PE*ij*, *i* = 0, 1, 2…, 100}MDAPE*j* (%) = median {|PE*ij*|, *i* = 0, 1, 2…, 100}*i* = 0,1,2…,100 (min); *j* = 1, 2, 3…, 10 (simulation number).
Final MDPE and MDAPE values are calculated as the mean of MDPE*j* and MDAPE*j* across all simulations:MDPE (%) = mean {MDPE*j*, *j* = 1, 2, 3…,10}MDAPE (%) = mean {MDAPE*j*, *j* = 1, 2, 3…, 10}*j* = 1, 2, 3…, 10 (simulation number).

### 2.7. Analysis Method

Values generated by ExtendSim^®^ were aggregated and analyzed in Microsoft Excel (version 2505), and the evaluation metrics were calculated. Since the primary aim of this study was to characterize algorithm trends and behavior under modeled conditions, statistical hypothesis testing was not performed.

## 3. Results

### 3.1. Visual-Based Assessment

**Figure 5:**
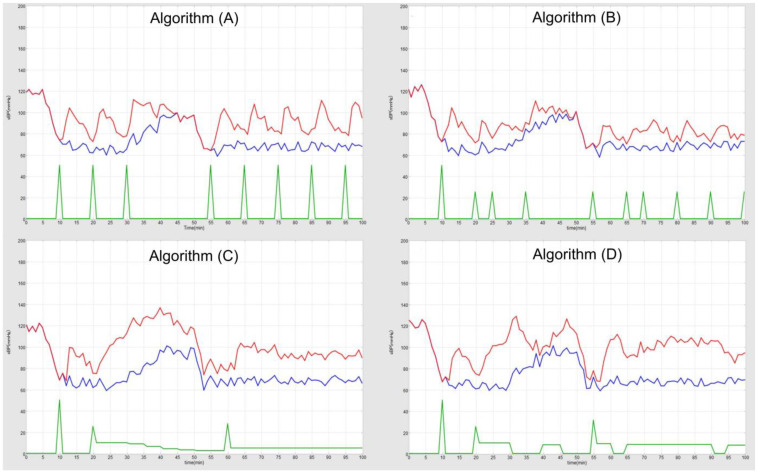
Example of a simulation run. The blue line represents the baseline sBP, the red line represents the generated sBP, and the green line represents vasopressor administration. The *x*-axis indicates time (minutes), and the *y*-axis indicates blood pressure (mmHg).

An example of sBP fluctuations under the four algorithms is shown in Figure 5. Each algorithm was consistently triggered when the generated sBP dropped below the threshold, preventing prolonged abnormal hypotension.

**Figure 6:**
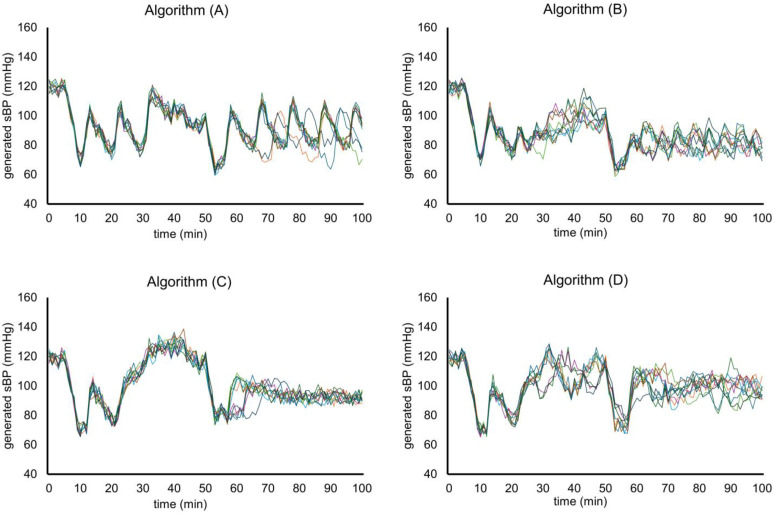
Generated sBP waveforms from ten simulation runs for each algorithm. The different colored lines represent individual simulation runs.

The generated sBP values over the 100 min simulation were recorded at each run. sBP transitions were visualized as waveform graphs, with fluctuations across ten runs per algorithm shown in Figure 6. Algorithm (A) exhibited large sawtooth-like fluctuations, while algorithm (B) showed smaller but still sawtooth-shaped variations. Algorithm (C), which incorporated continuous infusion, demonstrated more stable fluctuations than algorithms (A) and (B) and produced more frequent periods of elevated sBP. In contrast, algorithm (D) produced fewer elevated periods than (C) but larger fluctuations around the 85 mmHg threshold.

### 3.2. Metric-Based Assessment

**Figure 7:**
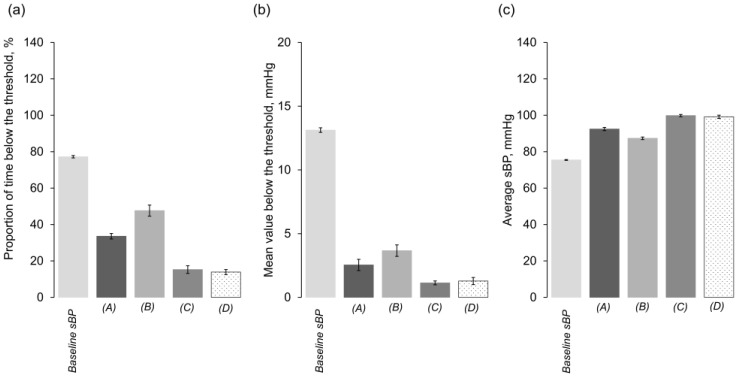
Graphs of each metric derived from five types of blood pressure data: baseline sBP and those generated by algorithms (A) to (D). (**a**) Proportion of time below the threshold. (**b**) Mean value below the threshold. (**c**) Average sBP. Panels (**a**–**c**) show the mean values calculated for each simulation run (*n* = 10). Error bars represent the standard deviation across runs.

The proportion of time below the threshold (PTBT) was 77.2 ± 0.7% for the baseline sBP, 33.6 ± 1.5% for (A), 47.6 ± 3.0% for (B), 15.2 ± 2.1% for (C), and 13.9 ± 1.4% for (D) (Figure 7a). These results indicate that algorithm-driven interventions based on lower-limit control substantially reduced the duration of hypotension. Regarding control efficacy, (A), which used repeated administration of a fixed vasopressor dose, enabled faster and more reliable recovery from hypotension compared with (B), which administered a half dose after the first occurrence. (C) and (D), both using continuous vasopressor infusion, further reduced PTBT, with (D) yielding slightly lower values than (C).

The mean value below the threshold (MVBT) was 13.1 ± 0.2 mmHg for the baseline sBP, decreasing to 2.5 ± 0.4 mmHg for (A), 3.7 ± 0.5 mmHg for (B), 1.1 ± 0.2 mmHg for (C), and 1.3 ± 0.3 mmHg for (D) (Figure 7b). These reductions demonstrate that lower-limit control not only shortened the duration below the threshold but also reduced the depth of hypotension. Consistent with PTBT, (A) was more effective than (B), while both (C) and (D) achieved further improvements. Notably, unlike PTBT, (D) showed a slightly higher MVBT than (C).

The average sBP was 75.4 ± 0.2 mmHg for the baseline sBP, increasing to 92.4 ± 0.9 mmHg for (A), 87.3 ± 0.7 mmHg for (B), 99.8 ± 0.6 mmHg for (C), and 99.2 ± 0.9 mmHg for (D) (Figure 7c). This metric reflects both the reduction in hypotension duration and depth as well as the impact of elevated blood pressure exceeding the threshold. Unlike PTBT and MVBT, where (A) and (B) exhibited higher values, (A) produced a higher average sBP than (B), while (C) and (D), both using continuous infusion, achieved greater increases. Although vasopressor administration was temporarily suspended when sBP exceeded 105 mmHg in (D), the average sBP over the entire observation period was similar to that of (C), where continuous infusion was uninterrupted.

Since the area under the threshold was greater than zero for all algorithms, we calculated the value as (average sBP + MVBT − threshold). The resulting values were 3.5 mmHg for the baseline sBP, 9.9 mmHg for (A), 6.0 mmHg for (B), 15.9 mmHg for (C), and 15.5 mmHg for (D). This metric represents the average blood pressure above the lower threshold achieved by each algorithm. Algorithms using continuous infusion showed higher pressures under supra-threshold conditions than those using repeated bolus alone. Temporarily suspending infusion when sBP exceeded 105 mmHg had minimal effect.

**Figure 8:**
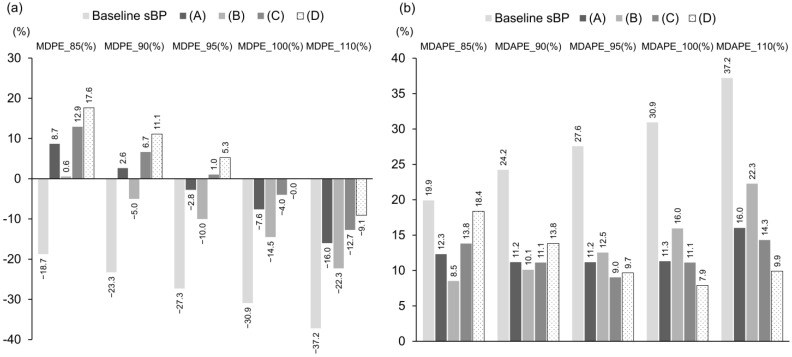
(**a**) MDPE at each target sBP level. (**b**) MDAPE values at each target sBP level. MDPE_X and MDAPE_X denote values when the target sBP was set to X mmHg (X = 85, 90, 95, 100, 110).

The MDPE and MDAPE values are summarized in Figure 8. As the target sBP increased, MDPE tended to decrease across all algorithms, indicating that MDPE is dependent on the designated target value. Similarly, MDAPE—although also influenced by the target pressure—showed that, when the target sBP was 95 mmHg or higher, algorithms incorporating continuous infusion (C) and (D) had lower MDAPE values compared with bolus-only algorithms (A) and (B), with (D) achieving less than 10%.

Since both MDPE and MDAPE are calculated relative to a specified target pressure, their values vary depending on the chosen target. Therefore, in control schemes such as lower-limit control, where the goal is to maintain blood pressure above a threshold rather than reach a specific target, these metrics may be unsuitable for evaluating the accuracy of vasopressor administration.

## 4. Discussion

### 4.1. Summary

This study used simulation to examine evaluation metrics for assessing the performance characteristics of vasopressor administration algorithms under a lower-limit control strategy.

### 4.2. Strengths and Reproducibility of the Simulation Framework

A major strength of this study is its standardized simulation framework, which enables reproducible comparisons of algorithm behavior under conditions that mimic clinical practice. As an example of a lower-limit threshold, we set sBP at 85 mmHg. This value, commonly used in clinical settings to guide decisions during rapid blood pressure fluctuations, was selected for convenience and does not necessarily represent the optimal threshold. The significance of this study lies in its ability to flexibly set thresholds and evaluate algorithm behavior under a variety of conditions. Extension to mean arterial pressure or individualized thresholds, both important indicators of organ perfusion, is also feasible.

The vasopressor algorithms implemented here were based on dosing patterns commonly used by anesthesiologists, reflecting realistic control behavior. Simulations could alternatively employ PID-based algorithms or AI-driven control strategies.

In this study, dosing adjustments were simulated at 5 min intervals, consistent with intermittent noninvasive blood pressure (NIBP) monitoring. While PID-based systems require continuous data, threshold-based control aligns with discrete monitoring and may be more applicable in clinical settings where continuous measurements are unavailable.

Simulation can also be applied retrospectively by estimating blood pressure trajectories from prior vasopressor dosing. Hypothetical scenarios can then be tested, allowing evaluation of algorithmic performance under conditions that cannot be ethically studied in patients.

### 4.3. Comparison with Previous Studies and Model Validity

Although prior studies have employed nonlinear dose–response models [18], the present simulation assumed a linear relationship between vasopressor dose and blood pressure change (ΔBP). This choice aimed to characterize algorithmic response patterns rather than replicate complex physiological mechanisms. The validity of the linear model is supported by three considerations: (1) the analysis window was not sufficiently long to elicit pharmacokinetic effects such as drug accumulation or metabolite formation; (2) a linear vasopressor effect of phenylephrine has been reported in patients with sepsis [19]. This example is not intended to limit the scope of the study to phenylephrine alone, but rather to illustrate the plausibility of a linear dose–response assumption; and (3) while many dose–response relationships are pharmacodynamically sigmoidal, the short simulation duration likely captures only the ascending linear segment of the curve. Moreover, in clinical practice, the rising phase of sigmoidal dose–response curves is often approximated linearly.

Previous research has widely examined automated vasopressor administration using PID control [10,12,13,14] and other feedback-based strategies [7,8,9], demonstrating strong control performance. In contrast, the present study focused on a threshold-based control strategy, emphasizing its structural simplicity, intuitive logic, and compatibility with intermittent blood pressure monitoring. Although this approach is less complex, consistent patterns observed across repeated simulations suggest that threshold-based control may adequately maintain blood pressure within acceptable ranges. Aligning dosing adjustment patterns with anesthesiologists’ common practices may also enhance interpretability.

### 4.4. Evaluation of Algorithms Under Lower-Limit Control

The goal of lower-limit control is to maintain blood pressure above a predefined threshold. Ideal performance corresponds to a PTBT of 0%. In our simulations, PTBT ranged from 14% to 48%, depending on the algorithm. Combining repeated bolus injections with continuous infusion reduced the duration of hypotension more effectively than bolus-only strategies.

MVBT reflected the depth of hypotension: bolus-only dosing produced larger fluctuations and higher MVBT, whereas continuous infusion produced smoother trends and lower MVBT. Even with similar PTBT, higher MVBT indicated deeper excursions below the threshold.

The average sBP captured the central tendency during the observation period. The sum of average sBP and MVBT, minus the threshold, indicated the average deviation above the threshold, offering insight into potential vasopressor overtreatment. Together, average sBP and MVBT provide meaningful indicators of control precision and overtreatment risk.

Conventional target-based metrics such as MDPE and MDAPE are less suitable for lower-limit control, where success is defined by maintaining pressures above a threshold rather than achieving a target value. Although non-parametric and resistant to outliers, MDPE and MDAPE do not intuitively reflect the magnitude of excursions or overall distribution. Furthermore, larger denominators (higher target pressures) can yield artificially lower errors, complicating comparisons across conditions.

### 4.5. Clinical Interpretation of Algorithms

The primary goal of this study was to characterize blood pressure fluctuations under different vasopressor administration strategies rather than to identify a superior algorithm.

PTBT was highest for algorithm (B), followed by (A), (C), and (D), suggesting that repeated moderate bolus doses enabled faster recovery from hypotension than smaller doses. Continuous infusion combined with bolus administration ((C) and (D)) further reduced hypotensive periods.

Vasopressor therapy is essential for managing intraoperative hypotension; however, excessive administration carries significant risks, particularly to organ perfusion [20,21,22]. To mitigate this, algorithm (D) incorporated temporary suspension of infusion when sBP exceeded 105 mmHg.

MVBT trends parallelled PTBT, with algorithms (C) and (D) showing lower values than (A) and (B). Algorithm (D) exhibited slightly higher MVBT than (C), suggesting a minor risk of exacerbated hypotension following vasopressor suspension. Average sBP was elevated in algorithms (C) and (D), reflecting improved hypotension avoidance. The combined metric of average sBP plus MVBT was similar between (C) and (D), indicating that suspending infusion above 105 mmHg offers limited additional benefit in reducing overtreatment. The slightly higher MVBT in (D) than in (C) suggests a potential risk of subsequent hypotension. Therefore, the clinical utility of this approach should be interpreted with caution.

### 4.6. Limitations

The results of this study should be interpreted within the constraints of its simulation framework. This study was entirely simulation-based and has not been clinically validated. The linear BP generation model simplified real pharmacodynamics, and baseline sBP variability was restricted to ±5 mmHg random error. Only one baseline BP pattern was applied. Clinical factors such as patient characteristics, concomitant medications, surgical procedures, and fluid therapy could not be directly investigated. Responses to hypotension were simulated using vasopressors alone, without fluid therapy. The vasopressor administration algorithm was designed as a simple rule-based approach, reflecting commonly used clinical practices based on 5 min intervals with NIBP monitoring. The algorithm did not incorporate pharmacokinetic or pharmacodynamic features such as time to peak effect. Although this study did not target any specific vasopressors, commercially available norepinephrine is supplied exclusively as salt formulations, with multiple salt types differing in molecular weight and potency [23,24]. In the blood pressure generation model used in this study, formulation type and its corresponding base equivalence were not considered. To ensure reproducibility, future simulation and clinical studies should clearly specify the formulation used (salt vs. base) and its base equivalence. These limitations do not undermine the interpretability of the evaluation metrics, but caution is required when extrapolating to clinical practice.

### 4.7. Future Directions

The management of intraoperative hypotension, which can influence patient outcomes, requires careful consideration of vasopressor algorithms and control accuracy, whether dosing is adjusted manually or via automated systems. Recent reports have highlighted the limitations of fixed-threshold blood pressure management and emphasized the importance of individualized approaches [5,25,26,27]. Strategies allowing permissive hypotension to minimize vasopressor use, such as vasopressor-sparing approaches, have also gained attention [21,22,28,29,30].

The simulation framework developed in this study enables flexible threshold settings and supports evaluation of algorithm performance under diverse hypotensive scenarios, making it suitable for preclinical assessment prior to clinical implementation. Moreover, the evaluation metrics introduced in this study are applicable to clinical research and provide a useful means of assessing lower-limit blood pressure control performance.

Future algorithm development is expected to incorporate vasopressor-specific characteristics and individualized approaches. The theoretical foundation and evaluation methodology presented in this study may support development and facilitate clinical implementation of more effective vasopressor strategies.

## Data Availability

Data available on request due to restrictions (e.g., privacy, legal or ethical reasons). Although certain model parameters and control functions (e.g., M(mg), f(t)) are part of an ongoing development effort and are not disclosed in detail, they were kept constant across all simulations to ensure consistency and reproducibility.

## Abbreviations

The following abbreviations are used in this manuscript:

BP	Blood pressure
sBP	Systolic blood pressure
PID	Proportional–integral–derivative
NIBP	Noninvasive blood pressure
PE	Percentage performance error
MDPE	Median performance error
MDAPE	Median absolute performance error
AUT	Area under the threshold

## References

[1] Wesselink E.M., Kappen T.H., Torn H.M., Slooter A.J.C., van Klei W.A. (2018). Intraoperative hypotension and the risk of postoperative adverse outcomes: A systematic review. Br. J. Anaesth..

[2] Gregory A., Stapelfeldt W.H., Khanna A.K., Smischney N.J., Boero I.J., Chen Q., Stevens M., Shaw A.D. (2021). Intraoperative hypotension is associated with adverse clinical outcomes after noncardiac surgery. Anesth. Analg..

[3] Walsh M., Devereaux P.J., Garg A.X., Kurz A., Turan A., Rodseth R.N., Cywinski J., Thabane L., Sessler D.I. (2013). Relationship between intraoperative mean arterial pressure and clinical outcomes after noncardiac surgery: Toward an empirical definition of hypotension. Anesthesiology.

[4] Monk T.G., Bronsert M.R., Henderson W.G., Mangione M.P., Sum-Ping S.T., Bentt D.R., Nguyen J.D., Richman J.S., Meguid R.A., Hammermeister K.E. (2015). Association between intraoperative hypotension and hypertension and 30-day postoperative mortality in noncardiac surgery. Anesthesiology.

[5] Futier E., Lefrant J.Y., Guinot P.G., Godet T., Lorne E., Cuvillon P., Bertran S., Leone M., Pastene B., Piriou V. (2017). Effect of individualized vs standard blood pressure management strategies on postoperative organ dysfunction among high-risk patients undergoing major surgery: A randomized clinical trial. JAMA.

[6] Stapelfeldt W.H., Khanna A.K., Shaw A.D., Shenoy A.V., Hwang S., Stevens M., Smischney N.J. (2021). Association of perioperative hypotension with subsequent greater healthcare resource utilization. J. Clin. Anesth..

[7] Sng B.L., Tan H.S., Sia A.T. (2014). Closed-loop double-vasopressor automated system vs manual bolus vasopressor to treat hypotension during spinal anaesthesia for caesarean section: A randomised controlled trial. Anaesthesia.

[8] Ngan Kee W.D., Khaw K.S., Ng F.F., Tam Y.H. (2013). Randomized comparison of closed-loop feedback computer-controlled with manual-controlled infusion of phenylephrine for maintaining arterial pressure during spinal anaesthesia for caesarean delivery. Br. J. Anaesth..

[9] Ngan Kee W.D., Khaw K.S., Tam Y.H., Ng F.F., Lee S.W. (2017). Performance of a closed-loop feedback computer-controlled infusion system for maintaining blood pressure during spinal anaesthesia for caesarean section: A randomized controlled comparison of norepinephrine versus phenylephrine. J. Clin. Monit. Comput..

[10] Joosten A., Alexander B., Duranteau J., Taccone F.S., Creteur J., Vincent J.L., Cannesson M., Rinehart J. (2019). Feasibility of closed-loop titration of norepinephrine infusion in patients undergoing moderate- and high-risk surgery. Br. J. Anaesth..

[11] Rinehart J., Lee S., Saugel B., Joosten A. (2021). Automated blood pressure control. Semin. Respir. Crit. Care Med..

[12] Joosten A., Delaporte A., Alexander B., Su F., Creteur J., Vincent J.L., Cannesson M., Rinehart J. (2019). Automated titration of vasopressor infusion using a closed-loop controller: In vivo feasibility study using a swine model. Anesthesiology.

[13] Ma M., Ho A., Joosten A., Rinehart J. (2022). In-silico analysis of closed-loop vasopressor control of phenylephrine versus norepinephrine. J. Clin. Monit. Comput..

[14] Rinehart J., Ma M., Calderon M.D., Cannesson M. (2018). Feasibility of automated titration of vasopressor infusions using a novel closed-loop controller. J. Clin. Monit. Comput..

[15] Nagata O., Morinushi E., Kuroyanagi A., Yasuma F. (2025). Development and evaluation of an automated phenylephrine delivery system by lower limit control for managing intraoperative hypotension. J. Anesth..

[16] Varvel J.R., Donoho D.L., Shafer S.L. (1992). Measuring the predictive performance of computer controlled infusion pumps. J. Pharmacokinet. Biopharm..

[17] Lee H.C., Ryu H.G., Jung C.W. (2017). Performance measurement of intraoperative systolic arterial pressure to predict in-hospital mortality in adult liver transplantation. Sci. Rep..

[18] Ngan Kee W.D. (2017). A random-allocation graded dose–response study of norepinephrine and phenylephrine for treating hypotension during spinal anesthesia for cesarean delivery. Anesthesiology.

[19] Flancbaum L., Dick M., Dasta J., Sinha R., Choban P. (1997). A dose–response study of phenylephrine in critically ill, septic surgical patients. Eur. J. Clin. Pharmacol..

[20] Lamontagne F., Marshall J.C., Adhikari N.K.J. (2018). Permissive hypotension during shock resuscitation: Equipoise in all patients?. Intensive Care Med..

[21] Endo A., Yamakawa K., Tagami T., Umemura Y., Wada T., Yamamoto R., Nagasawa H., Takayama W., Yagi M., Takahashi K. (2025). Efficacy of targeting high mean arterial pressure for older patients with septic shock (OPTPRESS): A multicentre, pragmatic, open-label, randomised controlled trial. Intensive Care Med..

[22] Roberts R.J., Miano T.A., Hammond D.A., Patel G.P., Chen J.T., Phillips K.M., Lopez N., Kashani K., Qadir N., Cairns C.B. (2020). Evaluation of Vasopressor Exposure and Mortality in Patients with Septic Shock. Crit. Care Med..

[23] Kotani Y., Belletti A., D’Andria Ursoleo J., Salvati S., Landoni G. (2023). Norepinephrine Dose Should Be Reported as Base Equivalence in Clinical Research Manuscripts. J. Cardiothorac. Vasc. Anesth..

[24] Wieruszewski P.M., Leone M., Kaas-Hansen B.S., Dugar S., Legrand M., McKenzie C.A., Turpin B.D.B., Messina A., Nasa P., Schorr C.A. (2024). Position Paper on the Reporting of Norepinephrine Formulations in Critical Care from the Society of Critical Care Medicine and European Society of Intensive Care Medicine Joint Task Force. Crit. Care Med..

[25] Bergholz A., Grüßer L., Khader W.T.A.K., Sierzputowski P., Krause L., Hein M., Wallqvist J., Ziemann S., Thomsen K.K., Flick M. (2025). Personalized Perioperative Blood Pressure Management in Patients Having Major Non-Cardiac Surgery: A Bicentric Pilot Randomized Trial. J. Clin. Anesth..

[26] D’Amico F., Kotani Y., Borello M., Colombo M., Rumore F., Papale F., Losiggio R., Landoni G. (2025). Reevaluating the Lower Limit of Renal Autoregulation: Does One Size Fit All?. Signa Vitae.

[27] Pang Z., Liang S., Zhou N., Zhu X., Guo Q., Sessler D.I., Zou W. (2025). Individualized blood pressure regulation and acute kidney injury in older patients having major abdominal surgery: A pilot randomized trial. Int. J. Surg..

[28] Lamontagne F., Richards-Belle A., Thomas K., Harrison D.A., Sadique M.Z., Grieve R.D., Camsooksai J., Darnell R., Gordon A.C., Henry D. (2020). Effect of Reduced Exposure to Vasopressors on 90-Day Mortality in Older Critically Ill Patients with Vasodilatory Hypotension: A Randomized Clinical Trial. JAMA.

[29] D’Amico F., Pruna A., Putowski Z., Dormio S., Ajello S., Scandroglio A.M., Lee T.C., Zangrillo A., Landoni G. (2024). Low versus High Blood Pressure Targets in Critically Ill and Surgical Patients: A Systematic Review and Meta-Analysis of Randomized Controlled Trials. Crit. Care Med..

[30] Guinot P.G., Martin A., Berthoud V., Voizeux P., Bartamian L., Santangelo E., Bouhemad B., Nguyen M. (2021). Vasopressor-Sparing Strategies in Patients with Shock: A Scoping-Review and an Evidence-Based Strategy Proposition. J. Clin. Med..

